# Clinical and prognostic significance of Hec1 expression in patients with Cervical Cancer

**DOI:** 10.3389/fonc.2024.1438734

**Published:** 2024-10-31

**Authors:** Yutai Zhao, Lei Xu, Cong Peng, Jie Deng, Chaolin Huang, Ling Lu

**Affiliations:** Department of Gynaecology and Obstetrics, Clinical Medical College and The First Affiliated Hospital of Chengdu Medical College, Chengdu, China

**Keywords:** cervical cancer, Hec1, independent risk factors, overall survival, progression-free survival

## Abstract

**Objective:**

Hec1 is a component of the Ndc80 kinetochore complex and is frequently upregulated in various cancers. However, the clinical significance of Hec1 in cervical cancer remains largely unknown. This study aimed to investigate the expression patterns of Hec1 in cervical cancer and its relationship with the clinicopathological characteristics of patients diagnosed with the disease.

**Methods:**

Immunohistochemistry was used to assess the expression of Hec1 in 136 cervical cancer tissue samples and 82 normal cervical tissue samples. The relationship between Hec1 protein expression and the clinicopathological characteristics of cervical cancer patients was analyzed using the Chi-square test. Additionally, the association between Hec1 protein expression and patient survival was examined using Kaplan-Meier survival curves. Independent risk factors affecting the prognosis of cervical cancer patients were analyzed using the Cox proportional hazards regression model.

**Results:**

The positive expression rate of Hec1 protein in cervical cancer tissues was 83.82%, significantly higher than the 7.31% in normal cervical tissues. Compared to patients with negative Hec1 expression, those with positive expression exhibited significantly higher FIGO staging, increased lymph node metastasis, greater depth of tumor stromal infiltration, and larger tumor diameter. Multivariable analysis using the Cox proportional hazards regression model indicated that Hec1 positive expression was an independent risk factor for both overall survival (HR = 2.79, 95% CI: 1.65–4.05, p = 0.012) and progression-free survival (HR = 1.81, 95% CI: 1.22-3.18, p = 0.002) in cervical cancer patients. Kaplan-Meier survival curve analysis showed that patients with positive Hec1 expression experienced a lower overall survival (HR: 2.72, 95% CI: 1.15–4.52, p = 0.004) and progression-free survival (HR: 3.12, 95% CI: 1.62–5.03, p = 0.002) when compared to those with negative Hec1 expression.

**Conclusion:**

Hec1 is significantly upregulated in cervical cancer tissues and associated with poor prognosis in cervical cancer patients. Therefore, Hec1 could be a novel biomarker, not only for the diagnosis and treatment evaluation of cervical cancer but also as an indicator for predicting the prognosis of cervical cancer patients.

## 1 Introduction

Cervical cancer ranks among the most common malignancies affecting women worldwide and is a significant cause of cancer-related deaths (1–3). The involvement of high-risk types of human papillomaviruses (HPV) in the onset of cervical cancer is well-documented; however, studies indicate that the presence of HPV alone does not inevitably lead to cancerous transformations (4–6). This suggests that additional genetic factors also play a crucial role in tumor development. The typical treatment for patients diagnosed with cervical cancer involves radiotherapy, chemotherapy, and surgery, which can often lead to remission (7). Nonetheless, a subset of patients experience relapse and succumb to disease progression. Furthermore, responses to treatment and clinical outcomes vary significantly among individuals, posing challenges to prognosis (8–10). Therefore, delving into the molecular mechanisms underlying cervical cancer and identifying markers that could aid in its early detection are crucial for improving both disease management and prognosis.

Hec1 is a component of the Ndc80 kinetochore complex and is frequently upregulated in various cancers (11–13). It serves as a critical component of the kinetochore’s outer layer and facilitates the spindle assembly checkpoint (14, 15). Hec1 is vital for establishing stable attachments between kinetochores and microtubules, ensuring accurate chromosome placement during cell division. Reduced levels of Hec1 disrupt chromosome alignment and lead to sustained activation of the spindle assembly checkpoint. Elevated expressions of Hec1 have been observed in a wide range of human malignancies, and correlate with poorer clinical prognoses in cancers including brain, liver, breast, colon, stomach, and lung cancers (11, 16–18). Although these findings suggest a significant role of increased Hec1 protein levels in cancer development or progression, the specific patterns of Hec1 expression and their implications in the context of cervical cancer have not yet been elucidated.

In this study, we investigated the expression patterns of Hec1 in cervical cancer tissues compared to healthy cervical samples using immunohistochemical techniques. Additionally, we explored the relationship between Hec1 expression status and the clinicopathological characteristics of patients diagnosed with cervical cancer. The findings from our research could improve the stratification of cervical cancer patients, potentially leading to more personalized initial treatments and helping to avoid either excessive or insufficient treatment for these individuals.

## 2 Patients and methods

### 2.1 Study population

This study was conducted with the approval of the Institutional Review Board at The First Affiliated Hospital of Chengdu Medical College (No. CYFY17243532) and adhered to the ethical principles outlined in the Declaration of Helsinki. This study involved a retrospective review of the medical records of 136 cervical cancer patients treated at The First Affiliated Hospital of Chengdu Medical College, a leading healthcare facility in Chengdu, China, between January 2018 and December 2019. Each patient’s staging was determined according to the International Federation of Gynecology and Obstetrics (FIGO) staging criteria for cervical cancer. The study’s inclusion criteria were as follows (1): histological confirmation of cervical cancer at any stage; (2) receipt of initial treatments such as radical hysterectomy with lymph node removal, concurrent chemotherapy and radiation therapy, radiation therapy alone, or chemotherapy at the study hospital; and (3) completion of treatment and availability of follow-up data. Exclusion criteria included: (1) incomplete treatment for any reason; (2) lack of adequate clinical and pathological records; and (3) presence of other cancers that could affect survival rates. For comparison, 82 normal cervical tissues were obtained from patients who underwent panhysterectomy due to uterine myoma.

### 2.2 Data collection and follow−up

Our department mandates lifelong monitoring of all cancer patients treated here, both for medical research purposes and to refine our treatment approaches. The follow-up protocol is structured with visits every three to six months for the first two years, followed by visits every six months for the next three to five years, and then annual visits from the sixth year onwards. We have dedicated staff assigned to carry out these follow-up activities, which were conducted through a combination of outpatient visits and phone communications. The follow-up period of this study ended in December 2023. Data were gathered from in-patient medical records, outpatient medical records, and records maintained by staff responsible for patient follow-ups. Overall survival (OS) is measured from the time of initial diagnosis to either death from any cause or the most recent follow-up. Progression-free survival (PFS) is calculated as the duration during which patients with cervical cancer survive without any disease progression or death following their treatment.

### 2.3 Immunohistochemistry

Sample tissues were immersed in a 4% solution of paraformaldehyde and left at room temperature for 48 hours. Subsequently, they were embedded in paraffin wax and sliced into 4 mm thick sections. These sections underwent dewaxing and dehydration, followed by antigen retrieval using a citric acid-based solution. The tissue sections were treated with 3% H_2_O_2_ and 10% goat serum to minimize endogenous peroxidase activity and nonspecific staining.

Next, the tissue sections were exposed to a primary antibody targeting Hec1, obtained from Abcam, for specific staining. The primary antibody was diluted at 1:200 and incubated overnight at 4°C. In the case of the negative control, this primary antibody application was replaced by using PBS. After a triple rinse in PBS, the sections were treated with an HRP-conjugated goat anti-mouse secondary antibody. Following three additional PBS washes, diaminobenzidine was applied to visualize the level of Hec1 expression. Pathologists evaluated the intensity of Hec1 expression using a Zeiss LSM500 microscope, manufactured by Zeiss International in Oberkochen, Germany.

Hec1 positivity was primarily determined by the presence of brown-yellow granules in the cell nucleus. Staining intensity was scored as follows: no staining received 0 points, weak staining (pale yellow) was scored 1 point, medium staining (brown-yellow granules) was 2 points, and strong staining (brown-black granules) was 3 points. Positive cell proportion scoring was categorized as follows: ≤5% positive cells received 0 points, 6% to 25% was 1 point, 26% to 50% was 2 points, 51% to 75% was 3 points, and 75% to 100% was 4 points. Finally, the two scores were multiplied together: a score >6 points indicated high expression, while ≤6 points indicated low expression.

### 2.4 Statistical analysis

We used SPSS statistical software to analyze the data. For continuous variables that followed a normal distribution, we employed the independent sample t-test to compare differences; for categorical variables, we utilized the Chi-square test or Fisher’s exact test. Patient survival was compared using Kaplan-Meier survival curves, and factors affecting patient prognosis were analyzed through multivariable analysis using the Cox proportional hazards regression model. We conducted Schoenfeld residual tests to verify that the proportional hazards assumption was satisfied before applying the Cox proportional hazards regression model. Differences were considered statistically significant when *p <*0.05.

## 3 Results

### 3.1 The expression pattern of Hec1 in cervical cancer tissues and normal cervical tissues

**Figure 1:**
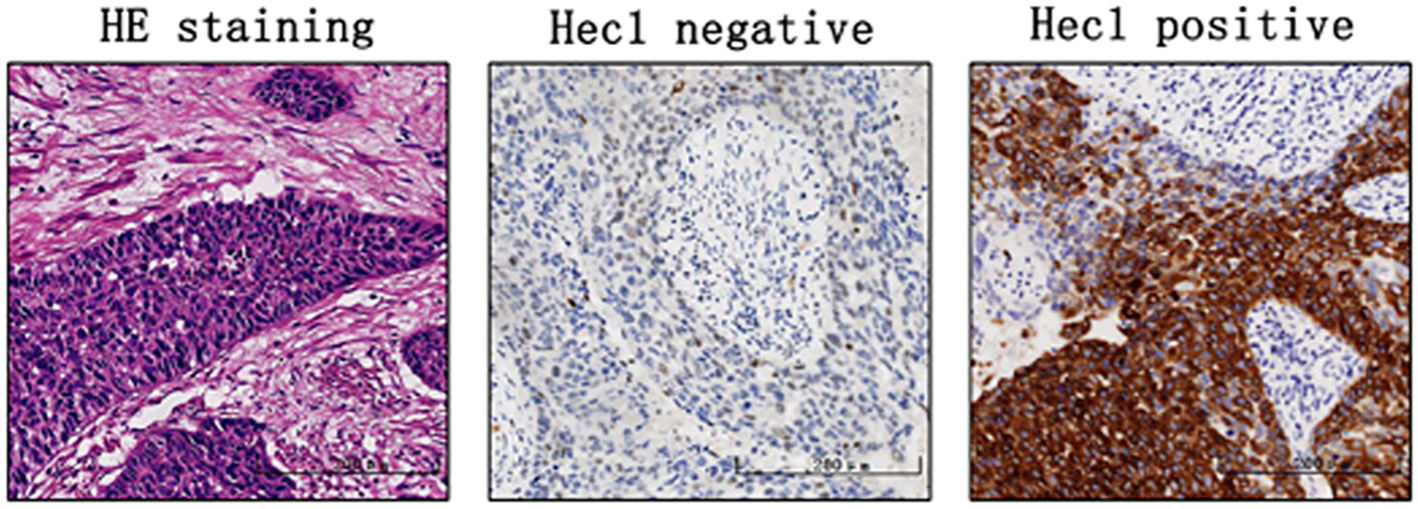
The Hec1 expression in cervical cancer tissues (×200).

Immunohistochemical analysis was conducted to assess Hec1 staining in 136 cervical cancer tissue samples and 82 normal cervical tissue samples. The findings indicated that Hec1 staining was positive in 83.82% of the cancerous tissues, whereas 7.31% of the normal tissues exhibited positive Hec1 staining (**Table 1** and **Figure 1**). This difference in Hec1 expression between cancerous and normal cervical tissues was statistically significant (p = 0.002).

**Table 1:** Hec1 expression in cervical cancer tissues and normal cervical tissues.

Sample types	Hec1 positive	Hec1 negative	Total	Positive rate(%)	*p-value*
Cervical cancer tissues	114	22	136	83.82	<0.0001
Normal cervical tissues	6	76	82	7.31	

Data were analyzed by Fisher’s exact test and A p<0.05 was considered statistically significant.

### 3.2 Relationship between Hec1 expression and clinicopathological characteristics of cervical cancer patients

The positive expression of Hec1 showed no statistical significance with the patient’s age, weight, histological types, or tumor differentiation. However, positive expression of Hec1 was significantly associated with tumor size, depth of tumor stromal infiltration, lymph node metastasis, and FIGO staging. Patients with positive expression of Hec1 demonstrated larger tumor size, greater depth of tumor stromal infiltration, increased lymph node metastasis, and higher FIGO stages (**Table 2**).

**Table 2:** Relationship between Hec1 expression and clinicopathological characteristics of cervical cancer patients.

Clinicopathological characteristics	Hec1 positive (114)	Hec1 negative (22)	*p-value*
Age (year)			0.489
<60	53	12	
≥60	61	10	
Weight (Kg)			0.249
<60	51	13	
≥60	63	9	
Histological types			0.322
Squamous cell carcinoma	80	15	
Non squamous cell carcinoma	34	7	
Tumor differentiation			0.165
Well	32	7	
Moderate	38	3	
Poor	44	12	
Tumor size (cm)			0.006
≤4	42	15	
>4	72	7	
Depth of stromal infiltration (mm)			0.001
≤3	45	17	
>3	67	5	
Lymph node metastasis			0.0002
Yes	27	14	
No	87	8	
FIGO staging			<0.0001
I-II	34	16	
III-IV	80	8	

Data were analyzed by Chi-square test and A p<0.05 was considered statistically significant and marked with bold text.

### 3.3 Investigation of prognostic factors for overall and progression-free survival in cervical cancer patients

To identify prognostic factors for cervical cancer patients, we employed univariate and multivariate Cox proportional hazards regression analyses. The univariate analysis revealed that tumor size, depth of stromal infiltration, lymph node metastasis, FIGO staging, and Hec1 expression were significant risk factors for overall survival rates (**Table 3**). When controlling for confounding variables such as age, tumor differentiation, tumor size, lymph node metastasis, depth of stromal infiltration, and FIGO staging, the multivariate analysis identified lymph node metastasis (HR = 1.76, 95% CI: 1.62-2.26, p = 0.001), FIGO staging (HR = 2.85, 95% CI: 1.73–4.16, p = 0.004), and Hec1 expression (HR = 2.79, 95% CI: 1.65–4.05, p = 0.012) as independent risk factors for overall survival rates (**Table 3**).

**Table 3:** Investigation of prognostic factors for overall survival in cervical cancer patients.

Characteristics	Univariate analysis	*p-value*	Multivariate analysis	Adjusted *p-value*
	HR (95%CI)		HR (95%CI)	
**Age (year)**	1.02 (0.87-1.06)	0.131	1.13 (0.94-1.21)	0.212
**Weight (kg)**	0.96 (0.82-1.13)	0.224	0.91 (0.85-1.09)	0.154
Histological types				
Squamous cell	1.00 (Reference)		1.00 (Reference)	
Non squamous cell	1.15 (0.92-1.23)	0.083	0.91 (0.82-1.24)	0.071
Tumor differentiation				
Well	1.00 (Reference)		1.00 (Reference)	
Moderate	0.92 (0.84-1.25)	0.215	0.87 (0.71-1.13)	0.102
Poor	1.04 (0.91-1.18)	0.321	1.16 (0.83-1.31)	0.115
**Tumor size (cm)**	2.79 (1.34 -3.54)	**0.014**	0.93 (0.67-1.39)	0.264
**Depth of stromal infiltration (mm)**	3.14 (2.52 -4.74)	**0.006**	1.14 (0.92-1.42)	0.178
Lymph node metastasis				
Yes	1.00 (Reference)		1.00 (Reference)	
No	0.74 (0.51-1.31)	**0.003**	0.76 (0.62-1.26)	**0.001**
FIGO staging				
I-II	1.00 (Reference)		1.00 (Reference)	
III-IV	3.27 (2.18-5.14)	**0.012**	2.85 (1.73-4.16)	**0.004**
Hec1 expression				
Positive	1.00 (Reference)		1.00 (Reference)	
Negative	0.81 (0.67-1.32)	**0.006**	0.79 (0.65-1.05)	**0.012**

Data were analyzed by a Cox proportional hazards regression model. CI, Confidence interval; HR, hazard ratio. The Multivariate analysis was adjusted for potential confounding variables, including age, weight, tumor differentiation, tumor size, lymph node metastasis, depth of stromal infiltration, and FIGO staging. A *p* < 0.05 was considered statistical significance and marked in bold text.

For progression-free survival rates, the univariate analysis indicated that tumor size, lymph node metastasis, FIGO staging, and Hec1 expression were significant risk factors (**Table 4**). After adjusting for confounders including age, weight, Histological types, tumor differentiation, tumor size, lymph node metastasis, and depth of stromal infiltration, the multivariate analysis identified tumor size (HR = 2.82, 95% CI: 1.32-4.04, p = 0.004), FIGO staging (HR = 3.64, 95% CI: 2.14-5.21, p = 0.007), and Hec1 expression (HR = 1.81, 95% CI: 1.22-3.18, p = 0.002) as independent risk factors for progression-free survival rates (see **Table 4**).

**Table 4:** Investigation of prognostic factors for progression-free survival in cervical cancer patients.

Characteristics	Univariate analysis	*p-value*	Multivariate analysis	Adjusted *p-value*
	HR (95%CI)		HR (95%CI)	
**Age (year)**	1.14 (0.92-1.17)	0.227	1.09 (0.87-1.32)	0.129
**Weight (kg)**	1.05 (0.76-1.24)	0.316	0.94 (0.81-1.22)	0.073
Histological types				
Squamous cell	1.00 (Reference)		1.00 (Reference)	
Non squamous cell	0.93 (0.81-1.16)	0.132	1.12 (0.84-1.31)	0.121
Tumor differentiation				
Well	1.00 (Reference)		1.00 (Reference)	
Moderate	1.15 (0.92-1.29)	0.082	0.93 (0.82-1.27)	0.175
Poor	0.95 (0.83-1.24)	0.152	1.23 (0.97-1.41)	0.304
**Tumor size (cm)**	3.17 (2.08 -4.32)	**0.003**	2.82 (1.32-4.04)	**0.004**
**Depth of stromal infiltration (mm)**	1.03 (0.87 -1.21)	0.214	0.82 (0.68-1.26)	0.218
Lymph node metastasis				
Yes	1.00 (Reference)		1.00 (Reference)	
No	0.73 (0.62-1.15)	**0.012**	0.94(0.81-1.28)	0.201
FIGO staging				
I-II	1.00 (Reference)		1.00 (Reference)	
III-IV	4.16 (2.35-6.05)	**0.005**	3.64 (2.14-5.21)	**0.007**
Hec1 expression				
Positive	1.00 (Reference)		1.00 (Reference)	
Negative	0.73 (0.62-1.04)	**0.017**	0.81 (0.72-1.18)	**0.002**

Data were analyzed by a Cox proportional hazards regression model. CI, Confidence interval; HR, hazard ratio. The Multivariate analysis was adjusted for potential confounding variables, including age, weight, tumor differentiation, tumor size, lymph node metastasis, depth of stromal infiltration, and FIGO staging. A *p* < 0.05 was considered statistical significance and marked in bold text.

### 3.4 Survival differences among cervical cancer patients based on Hec1 expression status

**Figure 2:**
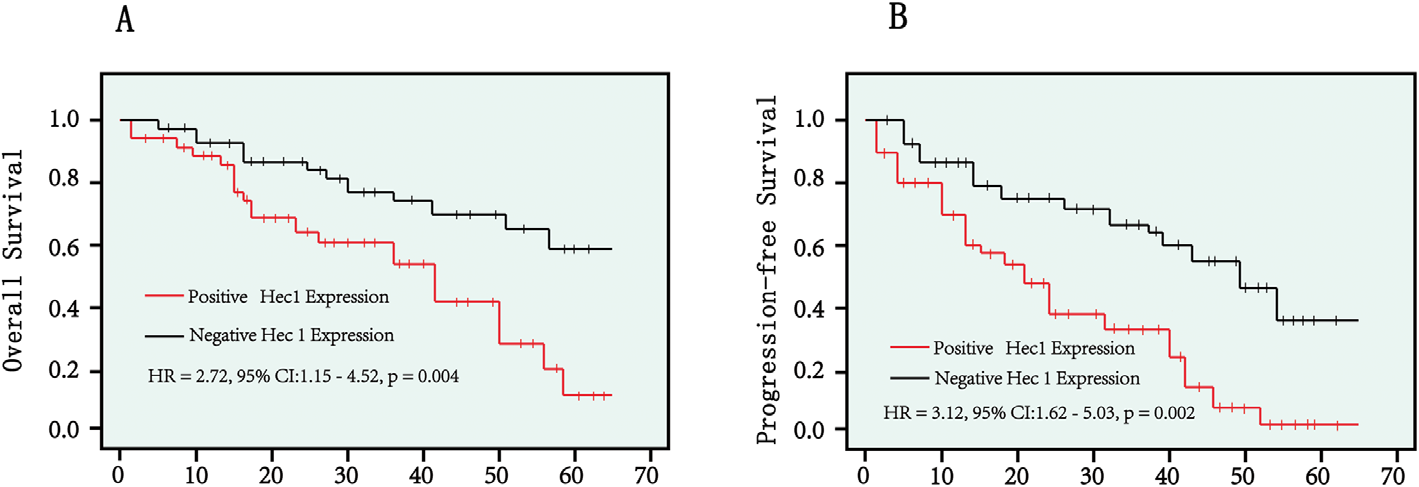
Kaplan–Meier curves were employed to compare overall survival rates **(A)** and progression-free survival rates **(B)** between patients with positive Hec1 expression and those with negative Hec1 expression. The log-rank test was utilized to determine the statistical significance between the two groups. A p < 0.05 was considered statistically significant.

The above findings demonstrated that Hec1 expression serves as an independent risk factor for both overall survival and progression-free survival in cervical cancer patients. We utilized Kaplan–Meier analysis to further investigate the survival disparity between patients with positive and negative Hec1 expression. The findings revealed that patients exhibiting positive Hec1 expression had a significantly reduced overall survival rate compared to those with negative Hec1 expression (HR: 2.72, 95% CI: 1.15–4.52, p = 0.004) (**Figure 2A**). Moreover, our analysis of progression-free survival indicated that patients with positive Hec1 expression experienced a lower progression-free survival rate than their negative Hec1 counterparts (HR: 3.12, 95% CI: 1.62–5.03, p = 0.002) (**Figure 2B**).

## 4 Discussion

Cervical cancer is a common malignant tumor in gynecology, posing a serious threat to women’s health (19, 20). In recent years, the incidence of cervical cancer has declined due to the promotion of cervical cancer vaccines and the widespread application of early screening technologies, which have led to increased rates of early diagnosis and treatment (21–23). However, for patients with metastatic and recurrent cervical cancer, the efficacy of traditional surgery and radiochemotherapy remains poor (24, 25). Therefore, it is particularly important to delve into the molecular pathogenesis of cervical cancer and to identify new diagnostic and therapeutic targets. This not only aids in the early diagnosis and treatment of cervical cancer but also enhances the evaluation of treatment effectiveness and the monitoring of tumor recurrence and metastasis.

This study found that the positive expression rate of Hec1 protein in cervical cancer tissues was 83.82%, significantly higher than the 7.31% in normal cervical tissues. This suggests that abnormal expression of Hec1 protein may play a key role in the development and progression of cervical cancer. Further research revealed that positive expression of Hec1 protein is associated with adverse clinical features in cervical cancer patients, such as increased FIGO staging, enhanced lymph node metastasis, deeper tumor stromal infiltration, larger tumor diameter, and reduced survival rates. Multivariable analysis using the Cox proportional hazards regression model further confirmed that Hec1 protein is an independent risk factor for poor prognosis in cervical cancer patients. Notably, these findings are consistent with other studies on the role of Hec1 protein in malignant tumors such as stomach cancer, bladder cancer, ovarian cancer, and liver cancer, where high expression of Hec1 protein is closely related to tumor occurrence, progression, and poor prognosis (11, 12, 16). Thus, the results of this study suggest that Hec1 may promote abnormal growth and differentiation of tumor cells, and its increased expression could enhance the tumor cells’ capabilities for invasion, migration, and metastasis, thereby facilitating tumor progression and poor prognosis.

Previous studies have shown that abnormally high expression of Hec1 plays a crucial role in tumorigenesis (26–28). Hec1 is a core component of the outer kinetochores and is closely associated with establishing correct microtubule attachments (14, 15). The loss of Hec1 at chromosome kinetochores prevents the normal recruitment of mitotic checkpoint proteins such as Mad1, Mad2, and Mps1 (29, 30). Past research has indicated that overexpression of Hec1 can lead to the abnormal activation of the mitotic checkpoint by upregulating and stabilizing Mad2 and Securin. Additionally, in mouse tumor models with overexpressed Hec1, tumor cells displayed abnormal chromosomal karyotypes, with a high occurrence of aneuploidy and tetraploidy, exacerbating chromosomal instability and thus promoting tumor formation (13, 27, 31). Furthermore, excessive expression of Hec1 can disrupt spindle assembly, leading to abnormal spindle formation, which can trigger the carcinogenic process. Most importantly, the overexpression of Hec1 can protect tumor cells from apoptosis triggered by abnormal chromosome segregation, thus promoting the unrestrained growth of tumor cells (26, 32).

Hec1 overexpression has been associated with the development of aneuploidy, a condition characterized by an abnormal number of chromosomes. Aneuploidy can promote tumor progression by increasing genetic diversity within the tumor, which in turn leads to the selection of more aggressive cancer cell clones (13, 27, 31). In the context of cervical cancer, this may explain the observed correlation between Hec1 overexpression and poor clinical outcomes, such as advanced FIGO staging, lymph node metastasis, and reduced survival rates. Moreover, tumor cells frequently evade apoptosis to survive and continue proliferation. Hec1 overexpression might protect cervical cancer cells from apoptosis induced by abnormal chromosome segregation, thereby supporting their unchecked growth (26, 32). This anti-apoptotic effect of Hec1 could further enhance the survival and dissemination of cancer cells, contributing to the poor prognosis observed in cervical cancer patients with high Hec1 expression.

Given its role in maintaining chromosomal stability and preventing apoptosis, Hec1 represents a promising therapeutic target. Recent studies have also found that inhibiting the expression of Hec1 in tumor cells can weaken the binding capacity between kinetochores and microtubules, thereby inducing cell cycle arrest and effectively inhibiting tumor growth (27, 33). Currently, studies have demonstrated that small molecule inhibitors INH1 and INH2 can target Hec1. These inhibitors can cause chromosomal misalignment during mitosis in breast tumor cells and disrupt the formation of normal spindles, with these mitotic abnormalities ultimately leading to the death of the breast tumor cells (17, 34). Although further research is necessary, these inhibitors hold potential for application in cervical cancer treatment, offering a novel approach to targeting cervical cancers characterized by high Hec1 expression.

The study has some limitations that should be acknowledged. The study population was from a single healthcare center in China, which may restrict the generalizability of our findings to other populations. The expression of Hec1 in cervical cancer can be influenced by a range of factors, including genetic diversity, environmental influences, lifestyle, and healthcare practices that may vary across different geographical regions and populations. Furthermore, variations in genetic polymorphisms, particularly in immune response genes, may contribute to the differential expression of Hec1 in cervical cancer patients from different ethnic backgrounds. Environmental factors, such as exposure to carcinogens, dietary habits, and socioeconomic status, could also impact Hec1 expression. Future studies would be beneficial to validate our findings in a more diverse population, including patients from different geographical regions and ethnicities. Such studies could provide a more comprehensive understanding of the role of Hec1 in cervical cancer and enhance the generalizability of our findings.

## 5 Conclusion

The expression of Hec1 in cervical cancer tissues is significantly higher than in normal cervical tissues. Cervical cancer patients with positive Hec1 expression had increased FIGO staging, lymph node metastasis, tumor stromal infiltration depth, and tumor diameter and poor prognosis. Furthermore, Hec1 is an independent risk factor affecting the prognosis of cervical cancer patients. Therefore, Hec1 could be a novel biomarker, not only for the diagnosis and treatment evaluation of cervical cancer but also as an indicator for predicting the prognosis of cervical cancer patients.

## Data Availability

The original contributions presented in the study are included in the article/supplementary material. Further inquiries can be directed to the corresponding authors.
